# *norCBD* disruption affects the H2-type six secretion system and multiple virulence factors in *Pseudomonas aeruginosa*

**DOI:** 10.3389/fmicb.2025.1717417

**Published:** 2025-12-19

**Authors:** Md Mahamudul Haque, Sara Badr, Kangmin Duan

**Affiliations:** 1 Department of Oral Biology, Rady Faculty of Health Sciences, University of Manitoba, Winnipeg, MB, Canada; 2 Children's Hospital Research Institute of Manitoba, Winnipeg, MB, Canada

**Keywords:** anaerobic adaptation, denitrification, DNR, nitric oxide, *norCBD*, *Pseudomonas aeruginosa*, type VI secretion system

## Abstract

The type six secretion system (T6SS) is a macromolecular weapon used by many Gram-negative bacteria. The T6SS functions as a needle injection system that delivers effector proteins directly into neighboring bacterial cells, thereby affecting their gene expression and physiological processes. *Pseudomonas aeruginosa* possesses at least three distinct T6SSs, designated as H1-, H2-, and H3-T6SS. Although extensive studies have been carried out on these T6SS systems in recent years, the regulatory mechanisms of T6SS remain incomplete. Here, we report the identification of *norCBD* as an operon that modulates the transcriptional activity of H2-T6SS. Both transposon insertion at *norCBD* and the deletion of the *norCBD* genes significantly reduced the CTX-*H2*-T6SS reporter activity. The *norCBD* operon encodes nitric oxide reductase (NorCBD), which reduces nitric oxide (NO) to nitrous oxide (N_2_O), a crucial step in reducing the toxic level of intracellular NO and facilitating anaerobic respiration. As the transcriptional regulator Dnr activates H2-type VI secretion system (H2-T6SS) in response to NO, experiments were carried out to examine whether *norCBD* deletion caused intracellular NO accumulation, which in turn disrupted Dnr-dependent regulation of H2-T6SS and virulence factors. The NO levels and Dnr-regulated gene expression were measured, and several virulence-related phenotypes were examined. The effects of NO donor sodium nitroprusside (SNP) and NO scavenger carboxy-phenyl-tetramethylimidazolineoxyl (CPTIO) were also tested. The data obtained indicate that deletion of *norCBD* led to intracellular NO accumulation, reduced H2-T6SS expression, and affected motility, pyocyanin production, and biofilm formation. Complementation of *norCBD* on a plasmid in the deletion mutant was able to restore H2-T6SS expression and the examined phenotypes to the wild-type levels. Treatment with CPTIO also restored H2-T6SS expression in the PAO1(Δ*norCBD*). These results indicate that NorCBD plays a critical role in maintaining NO homeostasis that is necessary for effective Dnr-mediated gene regulation and multiple virulence-related traits, highlighting the importance of redox balance in coordinating respiration and pathogenesis in *P. aeruginosa*.

## Introduction

*Pseudomonas aeruginosa* is a Gram-negative pathogen that plays a major role in chronic polymicrobial lung infections in cystic fibrosis (CF) patients (Nakatsuka et al., 2023). It is also a common cause of pneumonia, urinary tract infections, and burn wound infections. Antimicrobial-resistant *P. aeruginosa* has been recognized by the World Health Organization (WHO) as a critical priority pathogen that poses serious risks to global public health (Willyard, 2017).

The pathogenic success of *P. aeruginosa* is largely attributed to its arsenal of virulence factors (Qin et al., 2022). In particular, it employs specialized secretion systems to deliver toxins into host cells and competing microbes. Among these, the Type VI Secretion Systems (T6SSs) act as contractile nanomachines that mediate intercellular interactions by injecting toxic effectors into neighboring cells (Gallique et al., 2017). T6SSs have been implicated not only in interbacterial competition but also in metal ion acquisition (Chen et al., 2016; Cianfanelli et al., 2016; Wang et al., 2015) and biofilm formation (Alteri et al., 2013).

*P. aeruginosa* possesses three functionally distinct T6SSs: H1-T6SS, H2-T6SS, and H3-T6SS. H1-T6SS is primarily involved in intraspecies competition and delivers at least seven identified toxins (Tse1–Tse7) into rival bacteria (George et al., 2024; Hood et al., 2010). In contrast, H2-T6SS and H3-T6SS are capable of targeting both prokaryotic and eukaryotic cells (Lin et al., 2017). Despite their critical roles, the regulatory mechanisms that govern T6SS activity, particularly for H2- and H3-T6SS, remain incompletely understood.

To identify genes involved in H2-T6SS regulation, we employed transposon mutagenesis using a CTX-H2-T6SS reporter system. This screen revealed that disruption of the *norCBD* operon significantly decreased H2-T6SS reporter activity. The *norCBD* operon encodes the nitric oxide reductase complex (NorCBD), which catalyzes the reduction of toxic nitric oxide (NO) to nitrous oxide (N_2_O), a key step in anaerobic respiration.

The NorCBD complex is a cytochrome bc-type nitric oxide reductase. NorB forms the membrane-integrated catalytic subunit, NorC functions as a cytochrome c electron carrier, and NorD is thought to facilitate proper assembly and stabilization of the enzyme complex (Gao et al., 2016). Expression of the *norCBD* operon is activated by the nitric oxide-responsive transcription factor Dnr, which is itself regulated by Anr, the master transcriptional regulator that responds to low oxygen tension (Arai et al., 1997). Recent studies have shown that Dnr also controls the expression of H2-T6SS (Dang et al., 2022), which contributes to metal ion acquisition, interbacterial competition, and biofilm fitness in low-oxygen environments (Han et al., 2019; Wang et al., 2021).

Although Dnr activation is NO-dependent, the optimal intracellular concentration of NO for Dnr function is poorly defined. Excessive NO may impair Dnr activity or other NO-sensitive regulators, potentially through disruption of iron-sulfur clusters or heme groups (Crack et al., 2012, 2013; Fontenot et al., 2022). Based on this rationale and the observation of disrupted expression of H2-T6SS in *norCBD* mutant, we investigated, in this study, whether *norCBD* disruption might impair Dnr-mediated gene expression by allowing toxic NO accumulation, thereby altering regulatory outputs, including the activation of H2-T6SS.

While the role of NorCBD in NO detoxification has been well-documented (Zhou et al., 2019), its potential regulatory influence via modulation of intracellular NO levels and the resulting phenotypical changes have not been explored. We report the data suggesting that NorCBD not only facilitates anaerobic respiration but also contributes to the proper regulation of Dnr-dependent genes by maintaining intracellular NO balance that supports optimal Dnr activity.

## Materials and methods

### Bacterial plasmids and strains

Bacterial strains and plasmids used in all the experiments are shown in Table 1. *E. coli* and *P. aeruginosa* were grown on Luria-Bertani (LB) agar and broth at 37 °C. Different concentrations of antibiotics were applied. For *P. aeruginosa*, these concentrations were carbenicillin (250 μg/ml), trimethoprim (300 μg/ml), and tetracycline (70 μg/ml). The antibiotic concentrations used for *E. coli* were kanamycin (50 μg/ml), ampicillin (100 μg/ml), and tetracycline (12.0 μg/ml). Tetracycline (300 μg/ml) was used for constructing *P. aeruginosa* mutant PAO1(Δ*norCBD*), which was plated on a *Pseudomonas* isolation agar (PIA) plate.

**Table 1:** List of bacterial strains and plasmids used.

**Bacterial strains and plasmids**	**Characteristics**	**Sources/References**
***P. aeruginosa*** **strains**		
PAO1	Wild-type *P. aeruginosa*	Duan Lab
PAO1(Δ*norCBD*)	*norCBD* knockout mutant of PAO1	This study
PAO1(*ΔclpV3*)	*clpV3* knockout mutant of PAO1	Li et al., 2020
***E. coli*** **strains**		
DH5α	F– Φ80lacZΔM15 Δ(*lacZYA*-*argF*) U169 *recA1 endA1 hsdR17* (rK–, mK+) phoA supE44 λ- thi-1 *gyrA*96 *relA*1	Invitrogen
SM10-λ pir	Mobilizing strain, RP4 integrated into the chromosome; Km^r^	Duan Lab
**Plasmids**		
pEX18Tc	*oriT^+^ sacB^+^* gene replacement vector with multiple-cloning site from pUC18; Tc^r^	Hoang et al., 1998
pBT20	Mini-TnM delivery vector; Gm	Kulasekara et al., 2005
pAK1900	*E. coli-P. aeruginosa* shuttle cloning vector, Amp^r^	Sharp et al., 1996
pMS402	Expression reporter plasmid carrying the promoterless *luxCDABE*; Km^r^ Tmp^r^	Duan et al., 2003
CTX-6.1	Integration plasmid origins of plasmid mini-CTX- lux; Tc^r^	Kong et al., 2013
pRK2013	Broad-host-range helper vector; Tra^+^, Km^r^	Ditta et al., 1980
pEX18Tc-*norC* up	pEX18Tc carrying the upstream fragment of *norC*	This study
pEX18Tc-*norC*up+dw	pEX18Tc carrying the upstream and downstream fragment of *norC*	This study
pEX18Tc-*norD* up	pEX18Tc carrying the upstream fragment of *norD*	This study
pEX18Tc-*norD*up+dw	pEX18Tc carrying the upstream and downstream fragments of *norD*	This study
pAK-*norCBD*	pAK1900 with a 2874 bp fragment of *norCBD* between *KpnI* and *HindIII*; Amp^r^, Cb^r^	This study
CTX-*H2-T6SS*	Integration plasmid, CTX6.1 with a fragment of pKD-*hsiA2* containing *H*2 promoter (pMS402 containing *hsiA2* promoter region) region and *luxCDABE* gene; Kn^r^, Tmp^r^, Tc^r^	This study
CTX-*H2-T6SS* in PAO1(Δ*norCBD*)	PAO1(Δ*norCBD*) with the integration plasmid, CTX6.1 carrying *H2-T6SS* promoter region and *luxCDABE* (CTX-*H2-T6SS*) integrated in the chromosome; Kn^r^, Tmp^r^, Tc^r^	This study
CTX-*H2-T6SS* in PAO1(Δ*norCBD*)-*norCBD* in PAK1900	Integration plasmid, CTX6.1 with a fragment of pKD- *H2-T6SS-* PAO1(Δ*norCBD*)-*norCBD* in PAK1900 containing *H*2 promoter region and *luxCDABE* gene; Kn^r^, Tmp^r^, Tc^r^	This study

### Construction of H2-T6SS transposon mutagenesis library

For making a transposon mutagenesis library, the CTX-H2-PAO1 strain was subjected to mutagenesis by using a vector known as pBT20, with some modifications in the methods as described (Kulasekara et al., 2005; Liang et al., 2008). In detail, PAO1 was used as a recipient strain, whereas *E. coli* SM10 containing pBT20 was used as a donor. First, the overnight culture of the donor and recipient was scraped from the culture plate, and the OD_600_ was adjusted to 40 and 20 for the donor and recipient, respectively. Donors and recipients were taken in an equal ratio (20 μl). After mixing well, the culture was spotted on the LB agar plate and incubated at 37 °C for 3 h. Then, mixed cultures were taken and diluted. After that, a PIA medium with gentamycin (Gm; 150 μg/ml) was used for spreading purposes. Around 30,000 colonies were picked up and screened on 96-well plates, and a selective medium was used to make a transposon mutant library. Furthermore, colonies were incubated overnight in an LB medium containing selective antibiotics for the screening of genes that were involved in the expression of H2-T6SS. The next morning, the mutagenesis colonies were inoculated into 96-well plates, where the medium was LB (containing Gm at 50 μg/ml). For experimental purposes, both OD_600_ and luminescence were measured for 24 h at 37 °C. Colonies with altered expression (two or more) were selected for further screening. To avoid false positive results, a total of six additional repeats were done. Colonies with altered H2-T6SS expressions were confirmed and further characterized.

To confirm the transposon insertion site, we conducted an arbitrary primed polymerase chain reaction (PCR), and DNA sequencing was done for the PCR products as described, with some modifications (Liang et al., 2008). In brief, two primers, P7-1, which read out from one end of the transposon, and the arb1 primer, arbitrary (Table 2), along with the chromosomal DNA of the transposon mutant, were used for the PCR template for the first round. In the second round, another two primers, P7-2 and arb2 (Table 2), were used for the PCR. After the PCR products were purified by the Gel/PCR Extraction Kit (Geneaid, Taiwan). PCR products were further sequenced at the Sequencing Core Facility of the Research Institute in Oncology and Hematology (RIOH), Manitoba. Finally, a BLAST query was done to confirm the transposon insertion site.

**Table 2:** List of primers used.

**Primer**	**Sequence**	**Restriction site**
*norC-*up-S	TACGAATTCTGTTATACGCCGCAGGCTTG	*EcoRI*
*norC-*UP-AS	AAGGGATCCGCAGGATGAAGAACACACTC	*BamHI*
*norC-*dw-S	AAGGGATCCATCACCATCGACAAGGAGG	*BamHI*
*norC-*dw-AS	TCTAAGCTTGCGAAATCAGCATCCTCGAC	*HindIII*
*norD-*up-S	AAGGAATTCGCGGATGCTGAGCCTG	*EcoRI*
*norD-*up-AS	ATGGGATCCCGCTTCTTCTCCAGGTCG	*BamHI*
*norD-*dw-S	ATGGGATCCCGCTTCATCGACCTGGAG	*BamHI*
*norD-*dw-AS	ATGAAGCTTTCCCGAGTTGATCTGC	*HindIII*
pAK-*norCBD*-S	ATAGGTACCGTGCCGATGCGAACCCTGAC	*KpnI*
pAK-*norCBD*-AS	TATAAGCTTGCATGATGCTCTGGTTGCCG	*HindIII*
P-*norCBD*-F	GATCTCGAGTGGTCTACGTCCTGCAATGAG	*XhoI*
P-*norCBD*-R	CATGGATCCCAGGATGAAGAACACACTCC	*BamHI*
*H2-T6SS-*promoter-S	CGCCTCGAGCTATTACGCTCTAAATCAGC	*XhoI*
*H2-T6SS-*promoter-AS	TGAGGATCCCCTAACCCTTCAATGCACAC	*BamHI*
P7-1	CTAACAATTCGTTCAAGCCG	
arb1	GGCCACGCGTCGACTAGTACNNNNNNNNNNGATAT	
P7-2	GGATGCGTCTAAA AGCCTGC	
arb2	GGCCACGCGTCGACTAGTAC	

### Generation of a *norCBD* deletion mutant PAO1(*ΔnorCBD)* and a complementation strain

A deletion mutant of *norCBD* was generated in *P. aeruginosa* PAO1 using plasmid pEX18Tc (sucrose counter-selection system) (Hmelo et al., 2015; Hoang et al., 1998). In brief, both up and downstream fragments of the gene of interest (*norC*) were amplified by polymerase chain reaction using the designed primers shown in Table 2. Then, amplified fragments of up and downstream were cloned into a pEX18Tc vector that contains a counter-selectable *sacB* gene. After the double restriction enzyme digestion, both upstream and downstream products were ligated. PAO1(Δ*norC*) was obtained by tri-parental mating. Triparental mating was performed using the *E. coli* (helper strain) that contains pRK 2013, the donor strain *E. coli* had pEX18Tc-*norC* (up and down), and the recipient strain used for this purpose was PAO1. Overnight cultures of the donor, helper, and recipient strains were resuspended in a phosphate buffer saline (PBS). The bacteria were spotted on LB agar by mixing at a ratio of 2:2:1. An overnight culture was taken, and the developed pellets were resuspended in LB. The diluted bacteria suspension was spread on a PIA plate (containing tetracycline, 300 μg/ml) for the selection of merodiploid. For selecting a double crossover, the colony was streaked in LB agar containing 15% sucrose and no NaCl after the first crossover. Deletion mutants were verified by PCR using the confirmation primers (Table 2). Finally, PAO1(Δ*norCBD*) was constructed using the same method as PAO1(Δ*norD*) as a background and was confirmed by PCR.

Shuttle vector pAK1900 was used for the complementation of *norCBD* knockout mutants. First, the gene of interest (*norCBD*) was amplified using designed primers (pAK-*norCBD*-F and pAK-*norCBD*-R; Table 2). Then, the amplified product was cloned into a shuttle vector, pAK1900. Finally, the pAK-*norCBD* plasmid was transformed into PAO1(Δ*norCBD*) by electroporation.

### Construction of a promoter-reporter-based gene expression detecting system

The plasmid pMS402 is used for constructing a promoter-*luxCDABE* reporter because this plasmid contains a promoterless *luxCDABE* reporter gene cluster (Duan et al., 2003). First, the promoter region of *norCBD* was generated by PCR using a primer (P-*norCBD*-forward and P-*norCBD*-reverse) designed for the *norCBD* gene (Table 2) and inserted into the BamHI-XhoI site of vector pMS402 (upstream of promoterless *luxCDABE*) to generate plasmid pKD-*norCBD*. Second, pKD-*norCBD* was digested with PacI, and the larger band was isolated by Gel DNA Fragment Extraction Kit and cloned into CTX6.1, which was also digested with PacI to generate plasmid CTX- *norCBD*. Then, CTX- *norCBD* was transformed into *E. coli* SM10-λ *pir*. Finally, the *P. aeruginosa* reporter integration strain CTX-*norCBD* in PAO1 was obtained by bi-parental mating (Hoang et al., 1998). In brief, the process was performed using the *E. coli* SM10-λ *pir*, which contains CTX- *norCBD* and PAO1. Overnight cultures were resuspended in PBS buffer. The bacteria were spotted on LB agar by mixing at a ratio of 1:1. The overnight culture was taken, and the developed pellets were resuspended in Super Optimal Medium with Catabolic Repressor (SOC) medium. The diluted bacterial suspension was spread on a PIA plate (containing tetracycline) for selection. CTX-H2-T6SS in PAO1 and CTX-H2-T6SS in PAO1(Δ*norCBD*) were constructed using the method mentioned above.

### Measurement of promoter activities

Gene expression was measured by the lux-based reporter assays using a synergy plate reader (Biotek, USA) using the method as described (Duan et al., 2003). In detail, strains containing the reporter were grown overnight in liquid media (LB broth and M9 broth with supplements) and subcultured the next morning, and then diluted to an OD_600_ nm of 0.2. The diluted cultures were used as inoculants. After another 2 h of incubation, 5 μl of culture was inoculated into a 96-well flat-bottom plate containing 95 μl of fresh medium. A layer of 50 μl of filter-sterilized mineral oil (Sigma-Aldrich, USA) was applied on the surface to inhibit evaporation throughout the assay. Luminescence (counts per second, cps) was measured every 30 min for 24 h, and bacterial growth was monitored by measuring OD_600_ nm at the same time. The gene expression level was expressed by cps/OD_600_.

Sodium nitroprusside (SNP) stock solutions were prepared in PBS (pH 7.4). SNP was prepared and kept in the dark (Cai and Webb, 2020). The stock solution of SNP (7.5 mM) was filter sterilized (0.22 μm) and diluted into fresh M9 media with supplements of 20% glucose, 1M MgSO_4_, 1M CaCl_2_, and 0.5% casamino acid. During experimental procedures, SNP stocks were freshly prepared, placed on ice, and kept in dark conditions before use. The carboxy-phenyl-tetramethylimidazolineoxyl (CPTIO) stock solution was freshly prepared in HEPES buffer (0.4 mg/ml). The stock solution of CPTIO (100 mM) was also sterilized with a syringe filter and kept in ice and dark conditions (before use) due to light sensitivity. The strain containing the reporter was cultivated overnight in an M9 broth and then diluted to an OD_600_ nm of 0.2. After another 3 h of incubation, 5 μl of the bacterial cultures were inoculated into the parallel wells on a 96-well flat-bottom plate containing 95 μl of M9 medium and 10 μl SNP and CPTIO at different concentrations (Gao et al., 2022). In the test of the effect of NO donor and NO scavenger on H2-T6SS expression, NO donor, SNP (Sigma-Aldrich, USA), was added at different concentrations (10, 25, and 50 μM) in the media, and NO scavenger, CPTIO (Cayman Chemical, USA), was added at the concentrations of 2, 5, and 10 mM.

### Measurement of intracellular nitric oxide levels

The NO detection reagent diaminofluorescein-2 diacetate (DAF-2 DA; Sigma-Aldrich, USA) was used to quantify the cellular NO levels of *P. aeruginosa* bacterial cells using a previously established method (Su et al., 2014). In detail, to reach the exponential growth phase (OD_600_ = 0.2), the bacterial strain was subcultured for 3.5-4 h after being incubated overnight at 37 °C. Anaerobic bacterial growth was accomplished using the BD GasPak EZ Anaerobe Pouch System. The LB broth was supplemented with 50 mM KNO_3_ to promote anaerobic growth. The intracellular NO level was also measured under anaerobic conditions upon addition of 25 μM SNP to the LB broth. We also measured the intracellular NO concentrations under normoxic conditions, where strains were cultured in a 96-well plate. In brief, when the OD_600_ nm reached approximately 0.2, a 96-well plate with a flat bottom was filled with 180 μl of fresh M9 minimal media supplemented with 20 μl of bacterial culture and 50 μl of filtered (0.22 μm) mineral oil, and then allowed to grow inside the plate reader without or with the addition of SNP (25 μM). Following anaerobic and normoxic growth (at 5 h), 1 ml of shaking culture was incubated with 10 μM DAF-2 DA for 1 h at 37 °C. After incubation, the cell was washed with 1X PBS. Following washing, the fluorescence level of the reaction product DAF-2 T was measured (excitation wavelength, 495 nm; emission wavelength, 515 nm), normalized to OD600 using the FlexStation 3 Multi-Mode Microplate Reader (Molecular Devices, USA).

### Pyocyanin production measurement

Overnight culture (18 h) was used to extract and quantify the pyocyanin production, and the methods were described previously (Essar et al., 1990). In brief, the overnight culture was centrifuged, and 5 ml of supernatant was mixed with 3 ml of chloroform. One milliliter of 0.2 N HCl was added to the chloroform layer and centrifuged at 4,500 g for 10 min. After centrifugation, the upper layer (pink) was transferred to the cuvette, and absorbance was measured at 520 nm. The concentration of pyocyanin was expressed as micrograms produced per milliliter of culture supernatant, calculated by multiplying the extinction coefficient of 17.072 at 520 nm and expressed as micrograms per milliliter (μg/ml) (Mendoza et al., 2024; Montelongo-Martinez et al., 2023).

### Measurement of biofilm formation

Biofilm formation was measured by the protocol as described previously (O'Toole, 2011). Overnight cultured cells were inoculated into M63 medium (1:100 dilutions) containing glucose (0.2%), casamino acids (0.5%), and MgSO_4_ (1 mM), and 96-well microtiter plates were incubated at 37 °C for 24 h. The planktonic cell was removed after washing with 1X PBS (three times). Crystal violet solutions (100 μl) were added, and staining was allowed for each well at room temperature (15 min). All the wells were rinsed again with distilled water. One hundred and twenty five microliter acetic acid (30%) in water was dissolved in the remaining crystal violet on the plate. Finally, 100 μl solution was transferred into a new 96-well plate, and absorbance was measured at 550 nm.

### Detection of proteolytic activity

Proteolytic activity was measured using the method known as agar plate assay, as mentioned in the earlier studies, with some modifications (Gupta et al., 2009). Two microliters of cells from the overnight culture were plated on LB agar with 2% skim milk and incubated at 37 °C overnight. The presence of clear zones around the bacterial colonies suggests proteolytic activity, and the diameters of these zones were recorded.

### Motility assays

Bacterial motility activities were assessed according to the previously described protocol (Rashid and Kornberg, 2000). The medium for swarming motility contained glucose (5 g/l), agar (0.5%), and nutrient broth (8 g/l). The medium for swimming motility contained agar (0.3%), NaCl (5 g/l), and tryptone (10 g/l). Bacteria were spotted with 2 μl of the aliquot of the overnight LB culture for swarming and swimming motility assays. For swimming motility, the incubation was 15 h at 37 °C. For swarming motility, the incubation was at room temperature for 8 h, followed by incubation at 37 °C for 15 h. After inoculation, photographs were taken with a Vilber Lourmat Imager (Fusion FX7, Canada).

### Intracellular cAMP levels quantification

The intracellular cAMP levels were quantified using the cAMP Select ELISA kit (Cayman Chemical, USA). The cells from an overnight culture (OD_600_ was adjusted to 8) were collected by centrifugation, and 5 ml of culture was mixed with 500 μl of 0.1N HCl. The cells were allowed to undergo 30 s sonication (120 V). The bacterial cells were then centrifuged at 1,000 g for 10 min. After centrifugation, the supernatant was collected. The bacterial sample and standard were prepared according to the manufacturer's protocol and loaded into a 96-well plate supplied by the manufacturer. The 96-well plate was then covered with a plastic film and placed in an orbital shaker at 150 rpm under dark conditions (for 90 min). Using a plate reader, the absorbance was read at around 405 and 420 nm. The optical density of the bacterial sample concentration was determined from the standard curve plot using the equation as per the manufacturer's guidelines.

### Statistical analysis

All experimental results are presented as the mean ± standard error of the mean (SEM) from three independent experiments. Statistical significance tests and analysis were performed by using GraphPad PRISM version 10 (GraphPad Software, USA). Two-way analysis of variance (ANOVA) was applied to compare more than three groups with two independent variables. One-way ANOVA was used to compare more than three groups with one independent variable. Wherever applicable, significance levels are indicated as follows: ns (not significant, *p* > 0.05), ^*^p < 0.05, ^**^p < 0.01, ^***^p < 0.001, and ^****^p < 0.0001.

## Results

### Screening of genes affecting H2-T6SS by transposon mutagenesis

*P. aeruginosa* has three homologous yet functionally distinctive T6SS systems, H1-T6SS, H2-T6SS, and H3-T6SS, with H1-T6SS being the most studied. In the search for H2-T6SS regulators, a transposon mutant library was constructed to identify the regulatory pathway of T6SS. A transposon mutagenesis was performed in PAO1 harboring the CTX-H2-T6SS*-lux* reporter integrated on the chromosome by utilizing the mariner transposon vector pBT20 as previously described (Kulasekara et al., 2005; Liang et al., 2008). Approximately 30,000 colonies grown on selective media containing gentamicin were screened for altered H2-expression. Colonies with changed light production under an imager were selected for further studies. Arbitrary PCR and sequencing were performed to identify the transposon insertion sites in these mutants, as previously reported (Liang et al., 2008). From this transposon mutant library, eight genes were identified whose transposon insertion affected the expression of H2-T6SS (Table 3). Among them, PA0523 (*norC*) and PA0525 (*norD*) are located in the same operon. *norCBD* operon encodes nitric oxide reductase (NorCBD), which reduces nitric oxide (NO) to nitrous oxide (N_2_O), a key step for reducing the toxic level of intracellular NO and anaerobic respiration (Arai, 2011; Hino et al., 2010).

**Table 3:** Potential regulators of *H2*-T6SS (Transposon mutants).

**Gene name or number**	**Insertion site**	**Protein description**	**Max fold-change^a^**
*PA0523*(*norC*)	582172	Nitric oxide reductase subunit C	−2.8
*PA0525* (*norD*)	585588	Probable denitrification protein	−2.8
*PA0337*(*ptsP*)	380328	Phosphoenolpyruvate-protein phosphotransferase ptsp	+2.5
*PA0961-0962*	1046969	Probable cold-shock protein	+3
*PA1689*	1840252	Conserved hypothetical protein	+3
*PA2929*	3285258	Hypothetical protein	−2
*PA3798*	4257397	Probable aminotransferase	−2.5
*PA4467*	4997059	Hypothetical protein	−2

^a^Max fold-change, the maximal ratio of expression between the mutant and the wild-type.

### *norCBD* disruption affects the expression of H2-T6SS

**Figure 1:**
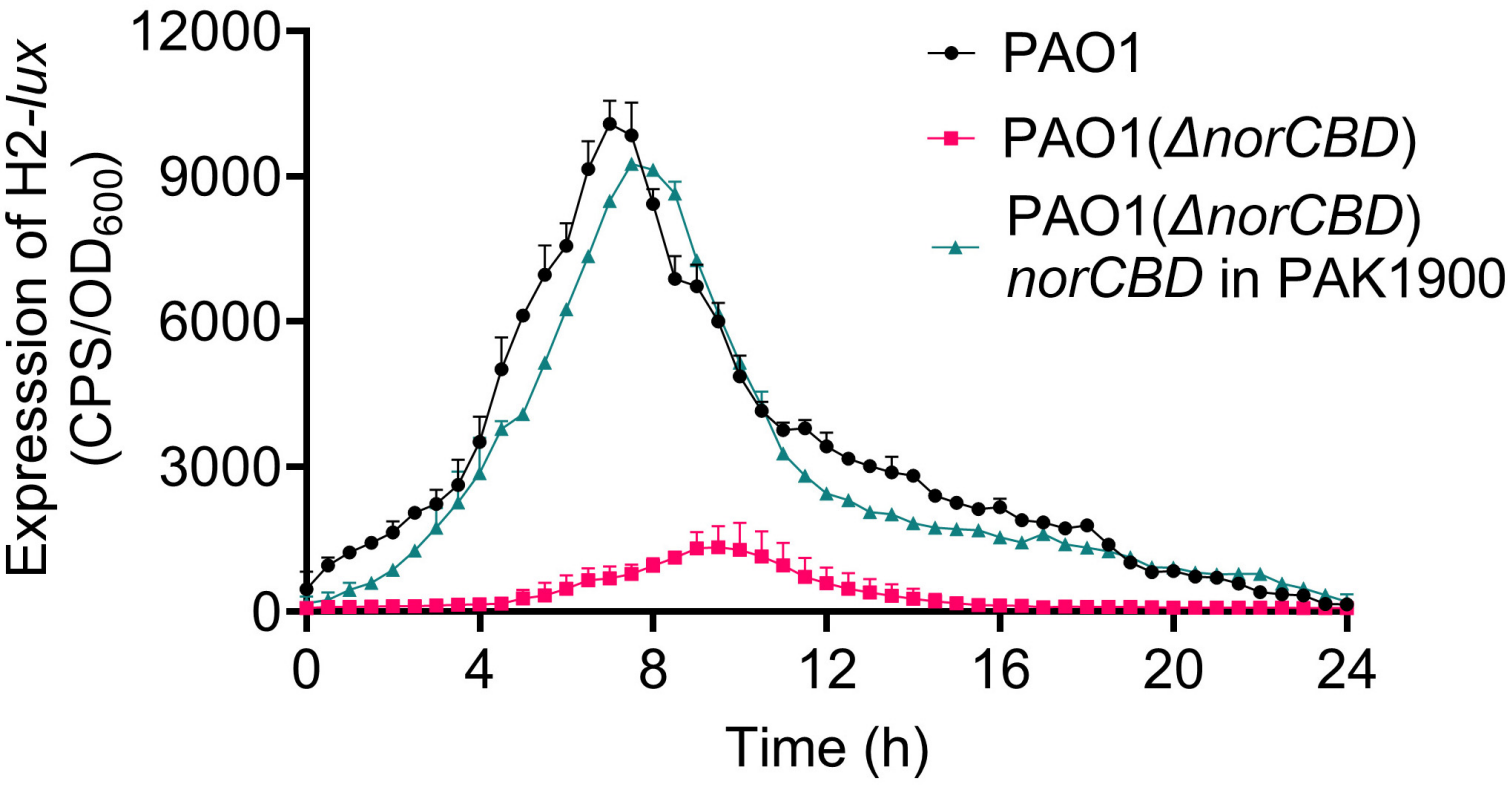
Deletion of *norCBD* resulted in decreased expression of H2-T6SS. The CTX-H2-T6SS reporter fusion integrated on the chromosome was used to measure the promoter activity of the H2-T6SS gene in PAO1(Δ*norCBD*). Compared to that in the wild-type PAO1, the promoter activity of H2-T6SS in PAO1(Δ*norCBD*) was significantly lower. The experiments were performed under normoxic conditions. The values represent the average of the triplicate experiments, and the error bars indicate standard deviations.

To verify the effect of *norCBD* disruption on the H2-T6SS expression, we constructed a *norCBD* deletion mutant PAO1(Δ*norCBD*) and compared the promoter activities of H2-T6SS in PAO1 and in the mutant. Same as the PAO1 harboring the CTX-H2-T6SS*-lux* reporter, the CTX-H2-T6SS*-lux* reporter was also chromosomally integrated in PAO1(Δ*norCBD*). A complementation strain of the mutant carrying the entire *norCBD* on a plasmid was also constructed. The transcriptional activities of H2-T6SS were measured and compared in these strains by measuring luminescence. Our results showed that the deletion of *norCBD* significantly reduced the CTX-H2-T6SS transcriptional activity (Figure 1), confirming the involvement of *norCBD* in the regulation of H2-T6SS. The maximal promoter activity of H2-T6SS at 8 h was around 8-fold lower in PAO1(Δ*norCBD*) compared with that in the wild-type PAO1. The complementation of *norCBD* restored the expression of H2-T6SS to the wild-type PAO1 level.

### Effect of NO donor and NO scavenger on the H2-T6SS expression

It is known that H2-T6SS is activated by Dnr in response to NO (Dang et al., 2022). It is expected that disruption of *norCBD* could increase the level of NO, which in turn would increase H2-T6SS expression. However, our results showed the opposite effect, namely, that potentially increasing NO in the mutant repressed H2-T6SS expression.

To test the possibility that the altered NO levels caused by the disruption of *norCBD* somehow contributed to the observed decrease of H2-T6SS expression in the *norCBD* mutant, we examined the expression of H2-T6SS in PAO1, PAO1(Δ*norCBD*), and the complementation strain in the presence and absence of NO donor, SNP.

**Figure 2:**
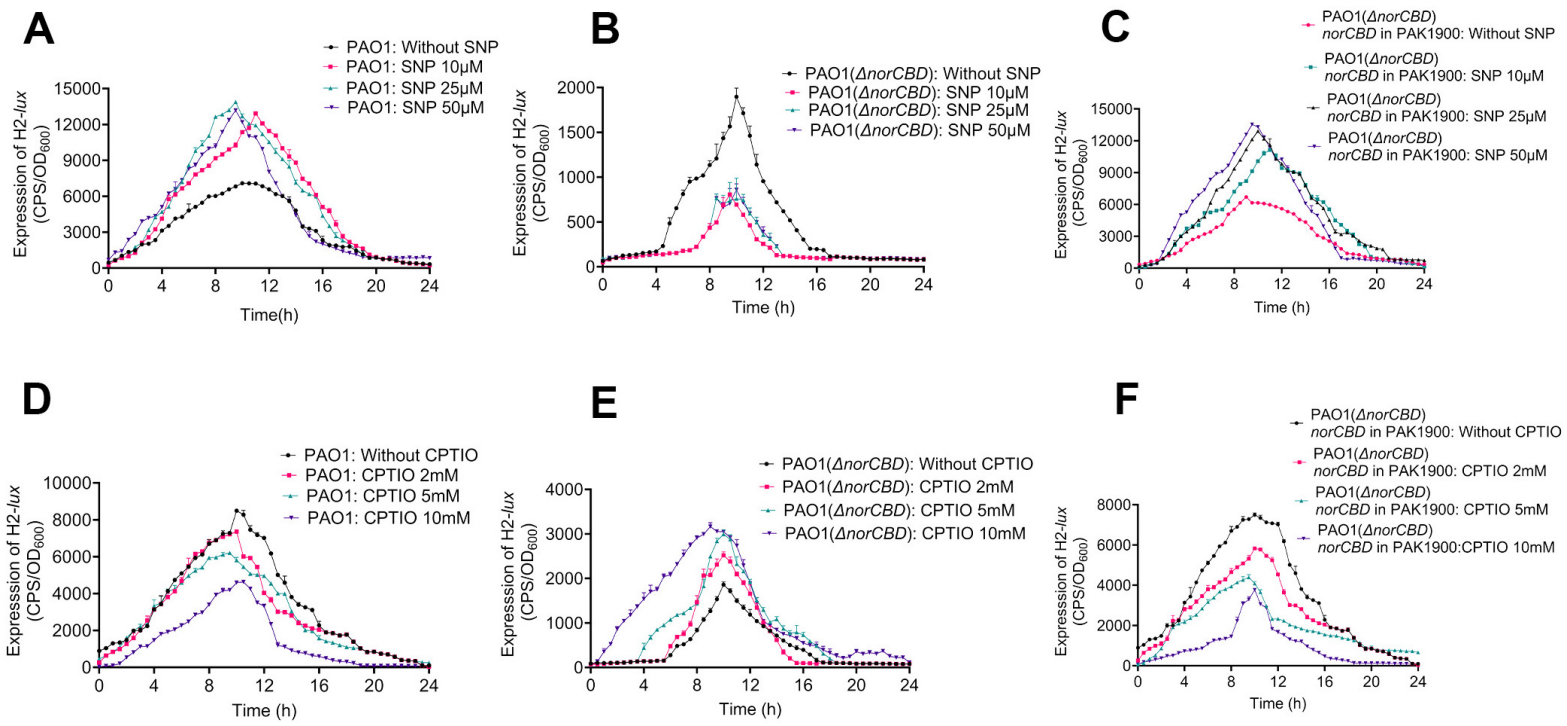
Effect of NO donor SNP and NO scavenger CPTIO on H2-T6SS expression: Different concentrations of SNP (10, 25, and 50 μM) and CPTIO (2, 5, and 10 mM) were added to the medium. **(A)** Our findings from this 24 h experiment demonstrated that the highest expression of H2-T6SS in PAO1 was achieved after 10 h, with the highest expression of the SNP at a dose of 25 μM, indicating the use of NO. **(B)** When compared to the expression level without SNP, H2-T6SS expression was considerably lower for the PAO1(Δ*norCBD*) strain. In comparison to the control, the lowest expression level was attained at a concentration of 50 μM SNP after 10 h. **(C)** Similar outcomes to those of the wild-type PAO1 were likewise displayed by the complementation strain of norCBD combined with CTX-H2-T6SS. Additionally, the experiment demonstrated that at a concentration of 25 μM SNP, the expression level peaked at 10 h. **(D)** Over time, the outcomes in wild-type PAO1 demonstrated a dose-dependent pattern. The expression of H2-T6SS gradually dropped when the concentration of CPTIO was raised to 5 mM and 10 mM, indicating that nitric oxide is being scavenged in the wild-type strains. When the H2-T6SS was examined, especially after 10 h, it was discovered that, in comparison to the control, the concentration of 10 mM revealed a low level of wild-type expression. **(E)** In the PAO1(Δ*norCBD*), when we increased the CPTIO concentration, the expression level increased considerably. After 10 h, the maximum expression was observed at CPTIO concentrations of 5 mM and 10 mM. **(F)** The complementation strain's H2-T6SS expression level coincides with that of the wild-type. Error bars indicate standard deviations.

As shown in Figure 2, the H2-T6SS expression was significantly changed following the addition of the SNP. However, opposite changes were observed in the *norCBD* mutant compared to the wild-type and the complementation strain. The expression of H2-T6SS was significantly increased in wild-type PAO1 compared to that in the absence of SNP (Figure 2A). The complementation strain of *norCBD* showed similar results to wild-type PAO1 (Figure 2C). In the *norCBD* deletion mutant, PAO1(Δ*norCBD*), a significant reduction in H2-T6SS expression was observed in the presence of SNP (Figure 2B). This observation contrasts with the increased H2-T6SS expression observed in the wild-type and complementation strains. These results appear to support our hypothesis that altered NO caused by *norCBD* disruption contributes to the changed H2-T6SS expression. In the wild type and the complementation strain, the increased H2-T6SS expression is probably a result of elevated Dnr activation in response to the increased NO level in the presence of SNP. In the *norCBD* mutant, however, further increase of NO on the already accumulated level potentially further impaired the Dnr-mediated H2-T6SS activation.

To further pursue this line of inquiry, the effect of NO scavenger CPTIO on the H2-T6SS expression was also determined. The transcriptional activity of CTX-H2-T6SS was similarly assessed in the wild-type PAO1, PAO1(Δ*norCBD*), and complementation strains in the presence and absence of NO scavenger CPTIO. CPTIO reacts rapidly and stoichiometrically with NO to form nitrogen dioxide (NO_2_), effectively removing NO from the cell (Goldstein et al., 2003; Lichtenberg et al., 2021). As shown in Figure 2, with CPTIO, the expression level of H2-T6SS was significantly reduced in wild-type PAO1 (Figure 2D) and in the complementation strain of *norCBD* (Figure 2F) in a dose-dependent manner. As anticipated, H2-T6SS expression was elevated in the PAO1(Δ*norCBD*) upon treatment with CPTIO (Figure 2E), indicating that NO scavenging alleviates the inhibition of Dnr activation caused by NO accumulation in the mutant.

### Accumulation of NO in the *norCBD* deletion mutant

**Figure 3:**
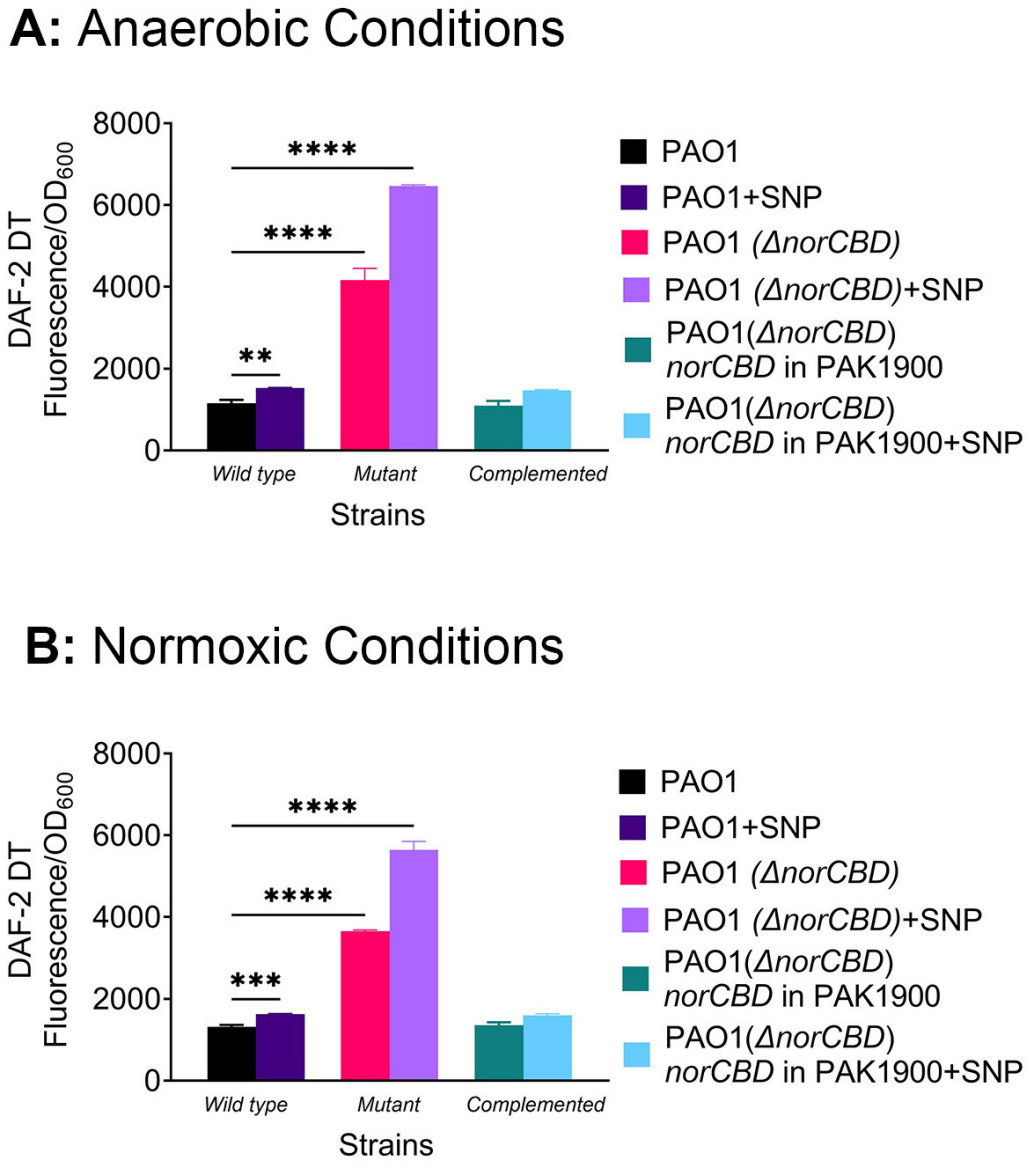
Comparison of intracellular nitric oxide levels. The level of intracellular nitric oxide in wild-type PAO1, PAO1(Δ*norCBD*) mutant, and the complemented strain was measured at the 5 h time point in the presence and absence of NO donor SNP. The NO levels reflected by the fluorescence intensity were normalized to OD_600_. **(A)** Under anaerobic conditions, the intracellular NO in PAO1(Δ*norCBD*) was 3.60-fold higher compared with that in the wild-type PAO1. Compared to the untreated, SNP treatment generated a 1.31-fold increase of NO in the wild-type, and a 1.55-fold change in the Δ*norCBD* mutant. The NO levels in the complementation strain were similar to those in the wild type. **(B)** Under normoxic conditions, the NO level was 2.78-fold higher in the Δ*norCBD* mutant compared to the wild-type. Upon the SNP treatment, the wild type showed a 1.23-fold increase of NO, lower than the 1.54-fold increase in the Δ*norCBD* mutant. Data was analyzed using one-way ANOVA. Error bars indicate standard deviations. **p < 0.01, ***p < 0.001, and ****p < 0.0001.

NO is a major signaling molecule in *P. aeruginosa*, but it is also toxic to the cells at high levels (Bogdan et al., 2000; Nisbett and Boon, 2016). To confirm the altered NO levels caused by the disruption of *norCBD*, we compared the level of intracellular NO in the wild type and the mutant strains, using diaminofluorescein-2 diacetate (DAF-2 DA), which is a cell-permeable fluorescent NO indicator converted to DAF-2 by the intracellular esterases. Upon reacting with NO, DAF-2 produces DAF-2 TA, a derivative that can be measured by fluorescence. The fluorescent intensity of DAF-2 TA gives a real-time measurement of NO. As expected, the *norCBD* deletion mutant showed significant accumulation of NO compared to the wild-type PAO1 (Figure 3A) under anaerobic conditions. At the early exponential phase (5 h), NO in the *norCBD* mutant was 3.60-fold higher than that in the wild-type PAO1. The complementation strains showed the accumulation of NO, which is close to wild-type PAO1.

As most of our H2-T6SS expression was measured in normoxic conditions, we also measured the intracellular NO concentrations in such conditions where the strains were cultured in a 96-well plate. Similarly, the results obtained (Figure 3B) showed that at 5 h, the intracellular NO level in the *norCBD* mutant was significantly elevated (2.78-fold) compared to the wild-type strain. The results confirm that NorCBD disruption causes an unchecked increase of intracellular NO, which likely results in dysregulation of H2-T6SS.

To compare how the wild-type and the mutant respond to NO disturbance, we also quantified intracellular NO levels following the addition of the NO donor, SNP. Final concentration of 25 μM was added to the wild-type, Δ*norCBD* mutant, and complementation strains, and NO levels were measured at the 5 h time point. Under anaerobic conditions, SNP treatment resulted in a 1.31-fold change of NO in the wild-type relative to untreated control, whereas the Δ*norCBD* mutant showed a higher fold change of 1.55 (Figure 3A). Similarly, under normoxic conditions, the wild type showed a 1.23-fold change, which was lower than the 1.54-fold change in the Δ*norCBD* mutant (Figure 3B). These results suggest that the presence of *norCBD* in wild-type effectively consumes excess NO, thereby preventing Dnr inactivation.

### Effect of *norCBD* disruption and NO accumulation on bacterial growth

It was noted that PAO1(Δ*norCBD*) exhibited slightly reduced growth compared to wild-type PAO1 and the complementation strain under anaerobic conditions (Supplementary Figure S1). We reasoned that the accumulation of NO and disruption of anaerobic respiration in the *norCBD* mutant affected the growth. To further investigate the impact of NO accumulation and denitrification disruption on the growth of the *P. aeruginosa*, we compared the growth of the *norCBD* mutant with that of the wild-type and complementation strain under normoxic conditions (Supplementary Figure S2). We also examined the effects of both an NO donor (Supplementary Figure S3) and an NO scavenger (Supplementary Figure S4) to determine whether modulation of NO levels could influence growth outcomes. Our results showed that there was no significant difference in growth between the wild-type and Δ*norCBD* mutant under normoxic conditions. A modest reduction of growth was only observed under anaerobic condition.

### Altered virulence traits in the *norCBD* mutant

*P. aeruginosa* is widely distributed in the environment and used as a paradigmatic model microorganism for studying virulence properties (Krell and Matilla, 2024). NO serves as a signaling molecule in *P. aeruginosa* (Barraud et al., 2009a). It reduces intracellular levels of cyclic di-GMP, a second messenger that promotes biofilm formation and regulates bacterial motility and influences quorum-sensing systems, which are associated with proteases in *P. aeruginosa* (Barraud et al., 2009b; Cai et al., 2020). Pyocyanin is one of the major virulence factors in *P. aeruginosa* and plays a crucial role in the pathogenesis of CF-related infections that cause chronic infections in the lungs, where NO is significantly associated with the production of pyocyanin (Gao et al., 2016). NO is endogenously produced in the denitrification pathway, linking it to metabolic and environmental sensing functions. To assess the broader effect of *norCBD* disruption, we tested several phenotypes in the *norCBD* mutant strain PAO1(Δ*norCBD*), together with PAO1 and complementation strains. The phenotypes tested include pyocyanin production, biofilm formation, proteolytic activity, and motility.

**Figure 4:**
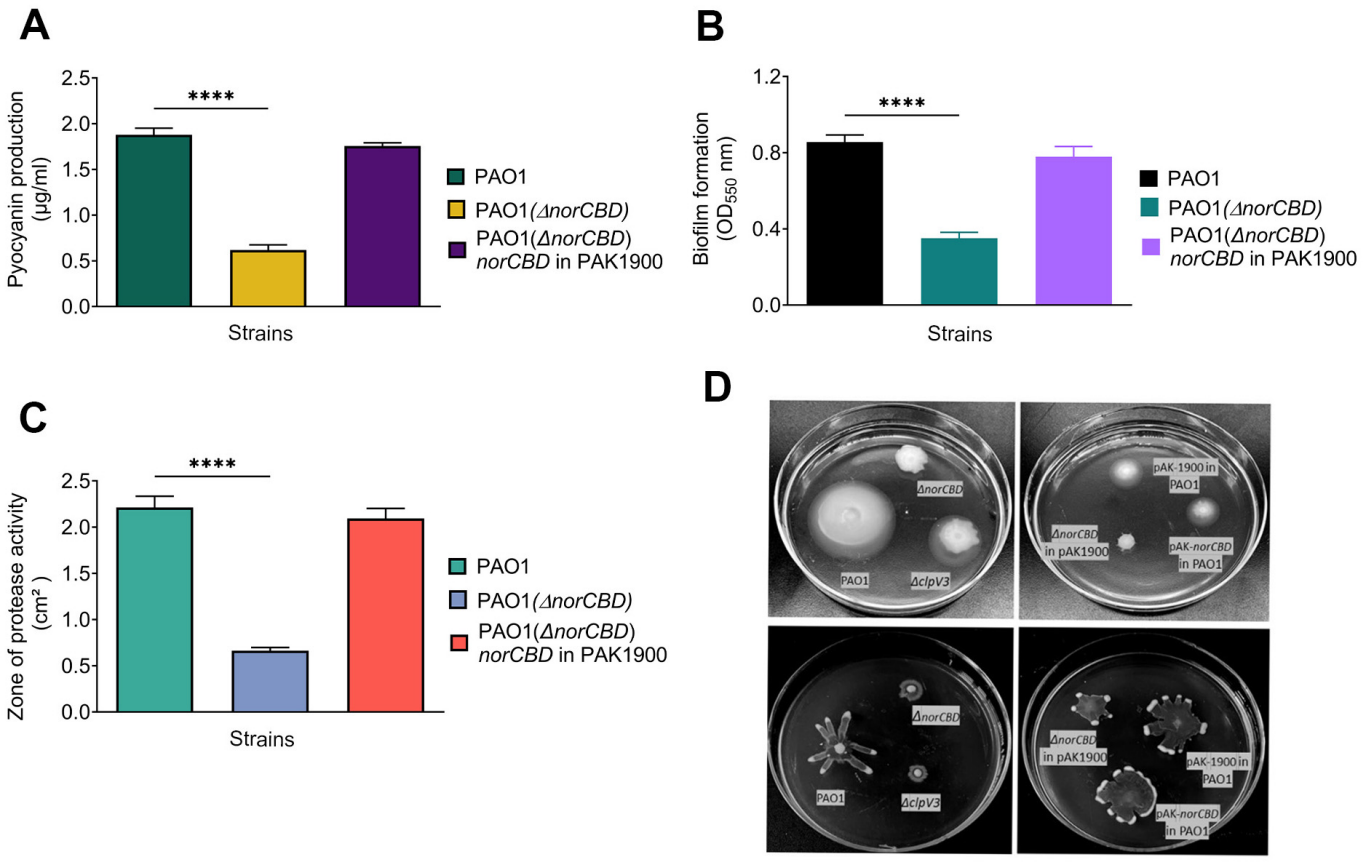
Multiple phenotypes of *P. aeruginosa* affected by the deletion of the *norCBD* gene. **(A–C)** Pyocyanin production, biofilm formation, and proteolytic zone were significantly decreased in PAO1(Δ*norCBD*) compared with PAO1 or the complementation strain. **(D)** Inactivation of *norCBD* results in reduced swimming and swarming motility. Data was analyzed using one-way ANOVA. Error bars indicate standard deviations. ****p < 0.0001.

As shown in Figure 4, when *norCBD* was inactivated, the level of pyocyanin was significantly reduced. The complementation of *norCBD* on a plasmid was able to restore the pyocyanin production to the wild-type level (Figure 4A). The biofilm formation assay showed that the PAO1(Δ*norCBD*) had a lower ability to form biofilms compared to the wild-type PAO1 (Figure 4B). Similarly, the *norCBD* mutant also showed reduced proteolytic activity (Figure 4C) and bacterial motility, especially the swimming motility and swarming motility (Figure 4D). These findings indicate that while NO is an important signal to activate multiple virulence-related phenotypes such as pyocyanin production, biofilm formation, proteolytic activity, and motility, the accumulation of NO in the PAO1(Δ*norCBD*) probably overrides its signaling function and results in the disruption of protease and pyocyanin production, biofilm formation, and motility. These results also indicate that increased intracellular NO and nitrosative stress might deactivate Dnr, which is a redox-sensitive regulator and interferes with signaling and quorum sensing, ultimately resulting in the regulation of these virulence traits.

### Deletion of the *norCBD* gene reduced levels of the second messenger cAMP

**Figure 5:**
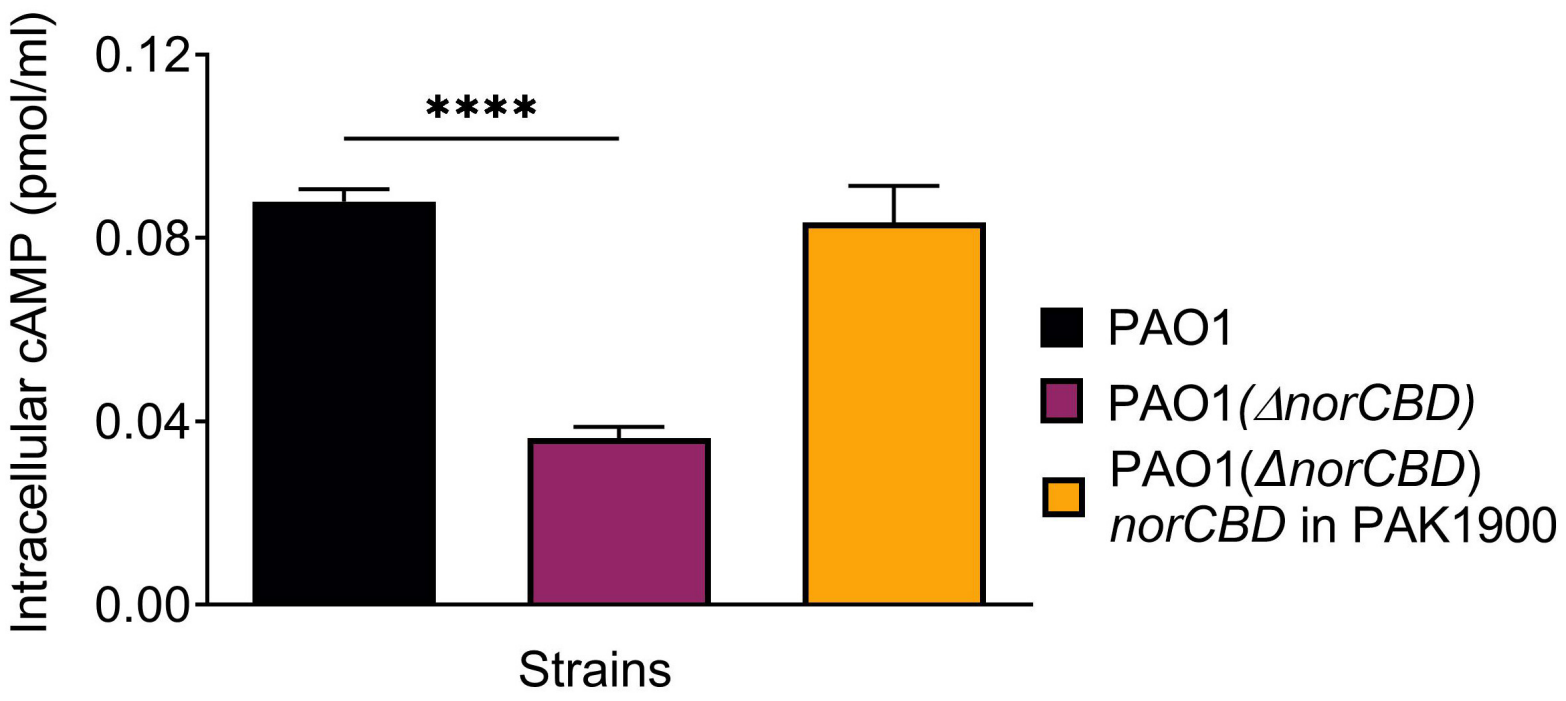
Reduced cAMP level in PAO1(Δ*norCBD*). cAMP concentration was quantified in wild-type PAO1, PAO1(Δ*norCBD*) mutant, and the complemented strain using the Cyclic AMP Select ELISA Kit. Data was analyzed using one-way ANOVA. Error bars indicate standard deviations. ****p < 0.0001.

It has been shown that the expression of H2-T6SS is positively regulated by NO-Dnr (Dang et al., 2022) but negatively modulated by cAMP-Vfr in *P. aeruginosa*. To investigate whether the observed phenotypical changes in the *norCBD* knockout mutant could be a result of altered cAMP levels, we compared the level of cAMP in the *norCBD* mutant with that of the wild-type and the complementation strains. Using the commercially available Cyclic AMP Select ELISA Kit, we assessed the intracellular concentrations of cAMP in PAO1 and PAO1(Δ*norCBD*), and the results obtained indicate that the disruption of *norCBD* reduced the cAMP level (Figure 5) when compared with the wild-type PAO1 and the complementation of *norCBD*. Considering the previous report that the expression of T6SS inversely correlates with cAMP levels (Zhang et al., 2022), these results could not explain the decreased H2-T6SS expression observed in the *norCBD* mutant. It is possible that the effect of the NO imbalance on H2-T6SS overrode the cAMP effect in the mutant.

## Discussion

This study identifies the *norCBD* operon as a previously unrecognized component that positively influences H2-T6SS transcription in *P. aeruginosa*. Deletion of *norCBD* elevated intracellular NO yet paradoxically suppressed H2-T6SS promoter activity; the expression was restored by genetic complementation and by NO scavenging with CPTIO, and H2-T6SS expression was enhanced by an NO donor (SNP) only in wild-type and complemented strains. The *norCBD* mutant showed a slight reduction in growth under anaerobic condition, with no significant differences observed in other conditions. It exhibited broad defects in pyocyanin production, proteolysis, biofilm formation, and motility. Together, these observations support a model in which NorCBD maintains an optimal intracellular NO range required for Dnr-dependent activation of H2-T6SS and associated virulence programs; excessive NO pushes cells outside this range, attenuating Dnr output and disrupting multiple traits.

The biphasic behavior of NO on H2-T6SS links two seemingly opposing effects: NO is the activating signal for the Anr-Dnr-H2-T6SS cascade (Luo et al., 2025; Wang et al., 2021), yet too much NO is inhibitory. In our data, moderate NO in the wild-type enhances H2-T6SS, whereas further NO elevation in the *norCBD* mutant depresses expression. Based on both the results observed and reports in the literature, we propose a potential model in which NorCBD in *P. aeruginosa* functions to maintain NO levels within a permissive range for Dnr function; without NorCBD, excessive NO would impair Dnr function.

This tentative model is supported by several lines of evidence. In the NO donor and scavenger experiments (Figure 2), we observed that the addition of donor SNP activated H2-T6SS expression, and the scavenger CPTIO decreased H2-T6SS expression in the wild-type. An activation of Dnr in response to NO (in turn, an activation of H2-T6SS) was expected when SNP was added, whereas CPTIO would result in decreased Dnr activity. However, in the *norCBD* mutant, opposite effects were observed, i.e., a decrease in H2-T6SS expression with SNP and an increase with CPTIO. With the proposed model, the impairment of Dnr function would only happen in the *norCBD* mutant but not in the wild type. In the wild-type, Dnr, in response to NO, induces *norCBD* expression, which in turn decreases NO accumulation, keeping the NO level in the Dnr functional range. The addition of the NO donor SNP in the wild type would increase NO level, allowing Dnr to activate H2-T6SS, and at the same time, Dnr induces *norCBD* expression, avoiding excessive NO accumulation. But in the *norCBD* mutant, the feedback loop is absent, and the NO donor would make NO accumulation even worse, resulting in further impairment of Dnr function and further decrease in H2-T6SS expression. Likewise, CPTIO would alleviate the impairment of Dnr caused by the excessive NO, increasing H2-T6SS expression in the mutant. Hence, this model appears to be supported by the data obtained from our study.

**Figure 6:**
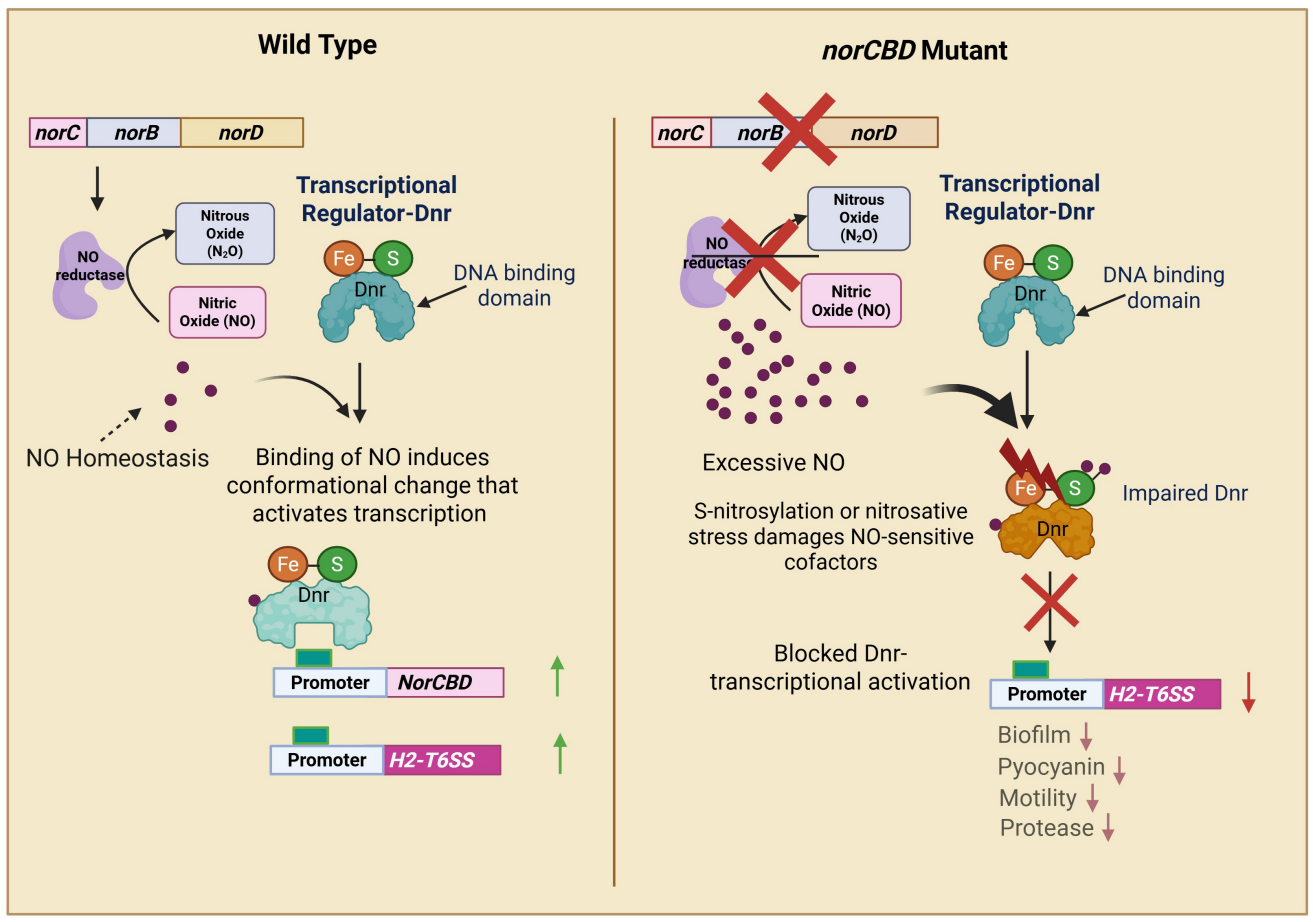
Schematic diagram outlining the role of NorCBD in H2-T6SS expression and multiple traits in *P. aeruginosa*. In the denitrification process under anaerobic conditions, nitric oxide reductase plays a key role. *norC, norB*, and *norD* are necessary for a functional nitric oxide reductase, which reduces NO to N_2_O. This process is significant as it protects the bacteria from the toxic effects of excessive NO. The active expression of *norCBD* and H2-T6SS is positively controlled by Dnr in response to NO. However, excessive NO may destabilize iron-sulfur clusters or heme groups, potentially affecting Dnr function and other NO-sensitive regulators. We propose a model where NorCBD keeps NO level in a permissive range for Dnr function; without NorCBD, excessive NO would impair the function of Dnr. In the wild type, Dnr, in response to NO, induces NorCBD production, which in turn decreases NO accumulation. Hence, in conditions of increased NO, such as the addition of the NO donor SNP, an increase in *norCBD* expression and other Dnr-regulated genes would be observed. But in the *norCBD* mutant, the feedback loop is absent, and NO accumulation would result in impairment of Dnr function and changed regulatory outputs, such as decreased H2-T6SS activation.

A possible explanation for the harming effect of excessive NO is that high NO nitrosylates or damages NO-sensitive cofactors such as Fe-S clusters or heme groups in Dnr and other regulators (Crack et al., 2013; Mettert and Kiley, 2014), reducing their activity despite NO being the initial activating signal. Dnr binds NO via its heme cofactor, leading to conformational changes that activate transcription of downstream genes (Giardina et al., 2008). However, the study also notes that excessive NO levels can lead to the formation of inactive Dnr complexes, suggesting a threshold beyond which NO becomes inhibitory. Another study reported that while low to moderate NO levels activate Dnr-dependent transcription, higher NO concentrations fail to do so, also implying that excessive NO impairs Dnr function (Castiglione et al., 2009). While NO is necessary for Dnr activation, an overabundance can lead to nitrosative stress, potentially modifying Dnr or its DNA-binding capability, thus inhibiting its function (Giardina et al., 2011). All these findings or suggested mechanisms appear to be in agreement with our tentative model (Figure 6).

The growth defect of the *norCBD* knockout mutant under anaerobic conditions is consistent with the enzyme's canonical role as the terminal NO reductase of denitrification (Arai, 2011; Zumft, 1997); without NorCBD, NO accumulates, respiration is compromised, and the resulting stress spreads into regulatory circuits that shut down non-essential systems, including T6SS. To verify that disruption of *norCBD* alters intracellular NO levels, we quantified NO using diaminofluorescein-2 diacetate (DAF-2 DA) in the presence and absence of NO donor, SNP. As expected, the Δ*norCBD* mutant exhibited a markedly higher NO accumulation than the wild-type PAO1 both in anaerobic and normoxic conditions. These results indicate that increased intracellular accumulation of NO and/or disruption in NO homeostasis occurs when *norCBD* becomes non-functional. The wild type displayed a limited increase in intracellular NO relative to the Δ*norCBD* mutant under both conditions upon SNP treatment when compared with the untreated condition, supporting the proposed model that functional NorCBD in the wild type effectively consumes excess NO and protects Dnr from impairment by the excessive NO.

Prior works have established that Anr and Dnr activate H2-T6SS genes and that H2-T6SS-linked effectors promote fitness and competition in low-oxygen niches (Luo et al., 2025; Wang et al., 2021). Our results corroborate this Dnr-dependent logic but add an upstream physiological layer: endogenous NO homeostasis, maintained by NorCBD, is necessary for this pathway. The relevant variable appears to be not NO presence *per se*, but its intracellular range. This agrees with broader observations that redox and nitrosative states interact with multiple global regulators in *P. aeruginosa* (Barraud et al., 2009b; Korner et al., 2003), including those that control acute-chronic transitions and metal acquisition, and suggests that H2-T6SS integrates respiratory status more tightly than previously appreciated.

The changes in virulence-related traits observed in our study support network-level coupling between denitrification physiology and global regulatory pathways. NO sensing in *P. aeruginosa* causes c-di-GMP-degrading phosphodiesterase to become active, which lowers intracellular c-di-GMP and promotes biofilm dispersal (Cutruzzola and Frankenberg-Dinkel, 2016). At low doses/level of NO induce biofilm dispersal and promote motility (Barraud et al., 2006; Howlin et al., 2017). As NO is known to reprogram second-messenger signaling, in particular, mis-timed or excessive NO can blunt c-di-GMP-driven switches that underlie biofilm formation and motility (Barraud et al., 2009b; Li et al., 2013). The potential connection of NO and Pyocyanin, a major virulence factor that is produced by 90–95% of the strains of *P. aeruginosa*, was demonstrated by Gao et al. (2016). The observation of this study reported that in the *nor* deletion mutant level of pyocyanin was reduced by 86% when compared with the wild-type PAO1 (Gao et al., 2016). Our results also showed that the level of PCN was effectively inhibited when we knocked out the *norCBD* gene, which indicates that the virulence properties of *P. aeruginosa* were highly affected by nitric oxide. The activation of *norCBD* gene thus assists the bacteria in successfully colonizing and establishing infection by producing pyocyanin. In addition, the denitrification process in *P. aeruginosa* is controlled by the quorum-sensing networks that regulate pyocyanin and protease production (Toyofuku et al., 2007). Through redox and NO-dependent signaling, NO metabolism affects QS-regulated virulence features, such as protease synthesis (Iiyama et al., 2017). Therefore, the reduced virulence-associated phenotypes (pyocyanin, biofilm biomass, proteolytic activity, and motility) in the Δ*norCBD* mutant are likely connected secondary consequences of impaired Dnr function and disrupted NO homeostasis.

We also observed a decrease in intracellular cAMP in the *norCBD* mutant. Although the cAMP-Vfr signaling axis coordinates diverse virulence-related phenotypes, including T6SS in *P. aeruginosa* (Fuchs et al., 2010; Zhang et al., 2022), the reduction in cAMP observed in the *norCBD* mutant does not explain the decreased expression of H2-T6SS genes. One possibility is that the dysregulation of NO in the mutant exerts an overriding inhibitory effect on H2-T6SS expression, masking any contributions from lowered cAMP levels. The observed H2-T6SS expression in the mutant was a result of the combined effects of different regulatory factors. The overall outcome from the opposing effects of NO dysregulation and decreased cAMP was a decrease in H2-T6SS expression. It is also possible that additional routes may exist in which the NO dysregulation can interfere with the cAMP-Vfr pathway. Future investigation is needed to fully understand the underlying mechanisms of the observed results.

In summary, this work identifies a regulatory layer upstream of Dnr, positioning NorCBD as a key component that enables Dnr-mediated activation of H2-T6SS across oxygen conditions. The results support a biphasic model in which H2-T6SS expression is maximal within an optimal NO window and falls when NO exceeds that range. Our findings broaden the scope of NO homeostasis from a purely anaerobic function to a principal factor that influences *P. aeruginosa* physiology across oxygen gradients, with NorCBD acting as an essential balancer for NO homeostasis. However, further studies are required to map the complex interactions among the NO, cAMP-Vfr, and c-di-GMP circuits.

## Data Availability

The original contributions presented in the study are included in the article/Supplementary material, further inquiries can be directed to the corresponding author.
