# Supplementary material for: norCBD disruption affects the H2-type six secretion system and multiple virulence factors in Pseudomonas aeruginosa
Source: Front Microbiol. 2025 Dec 19;16:1717417. doi: 10.3389/fmicb.2025.1717417 (PMC12757424; doi:10.3389/fmicb.2025.1717417)
Supplement: Supplementary file 1 [file Data_Sheet_1.docx]

Supplementary Material

***norCBD* disruption affects the H2-type six secretion system and multiple virulence factors in *Pseudomonas aeruginosa***

Md Mahamudul Haque^1^, Sara Badr^1^ and Kangmin Duan^1,2*^

1. Department of Oral Biology, Rady Faculty of Health Sciences, University of Manitoba, Winnipeg, Manitoba, Canada
2. Children's Hospital Research Institute of Manitoba, Winnipeg, Manitoba, Canada

*****For Correspondence**:** Email: [Kangmin.Duan@umanitoba.ca](mailto:Kangmin.Duan@umanitoba.ca)


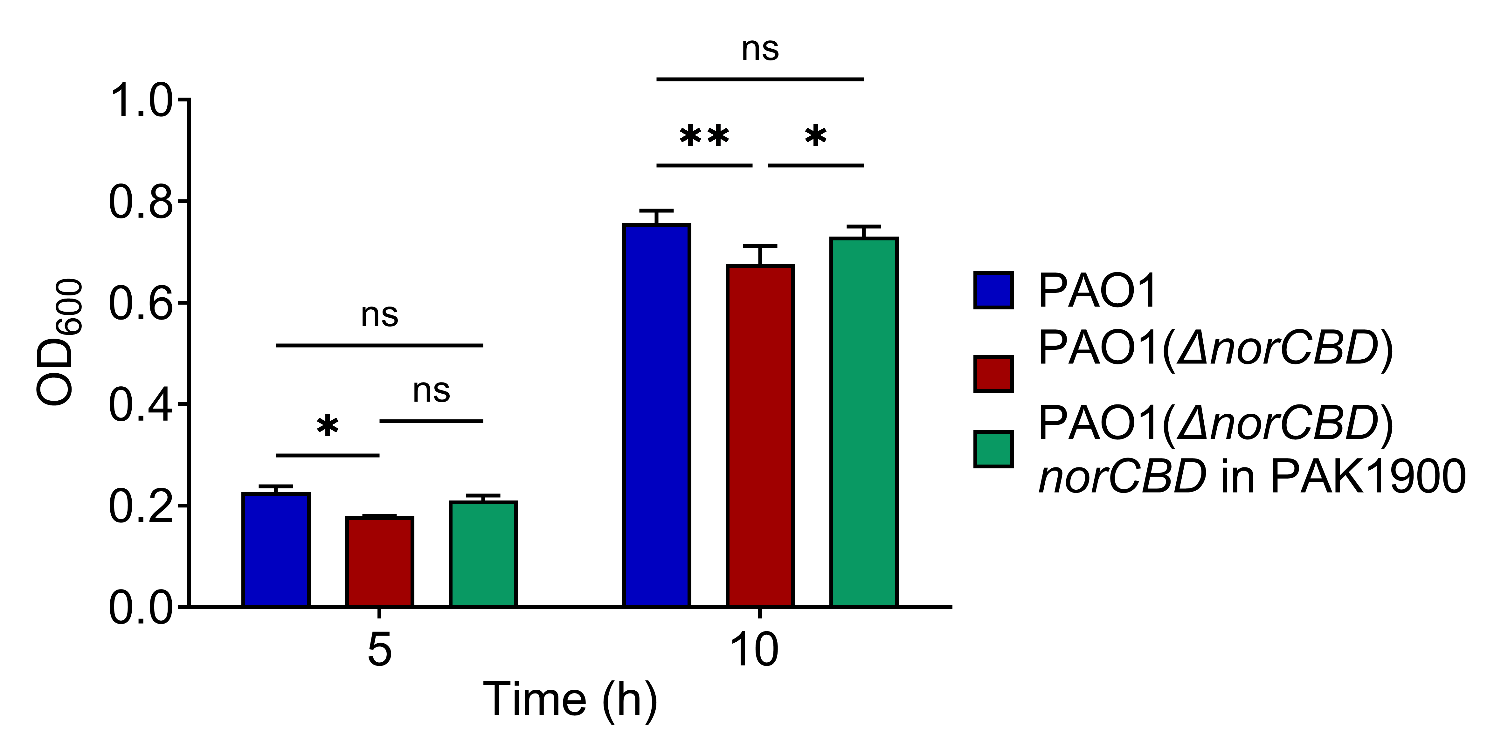


**Supplementary Figure 1. *P. aeruginosa* growth (OD_600_) under anaerobic conditions.** The growth of wild-type PAO1, PAO1*(ΔnorCBD),* and complementation strains, PAO1*(ΔnorCBD)-*norCBD in PAK1900, was measured at 5 h and 10 h under anaerobic conditions. Under anaerobic conditions, the growth is relatively slower in PAO1*(ΔnorCBD)* than in the wild-type PAO1 and the complementation strain. Data were analyzed using two-way ANOVA and Tukey's multiple comparisons test. Error bars indicate standard deviations. *ns* (not significant, *p* > 0.05), * *p* < 0.05 and ** *p* < 0.01.


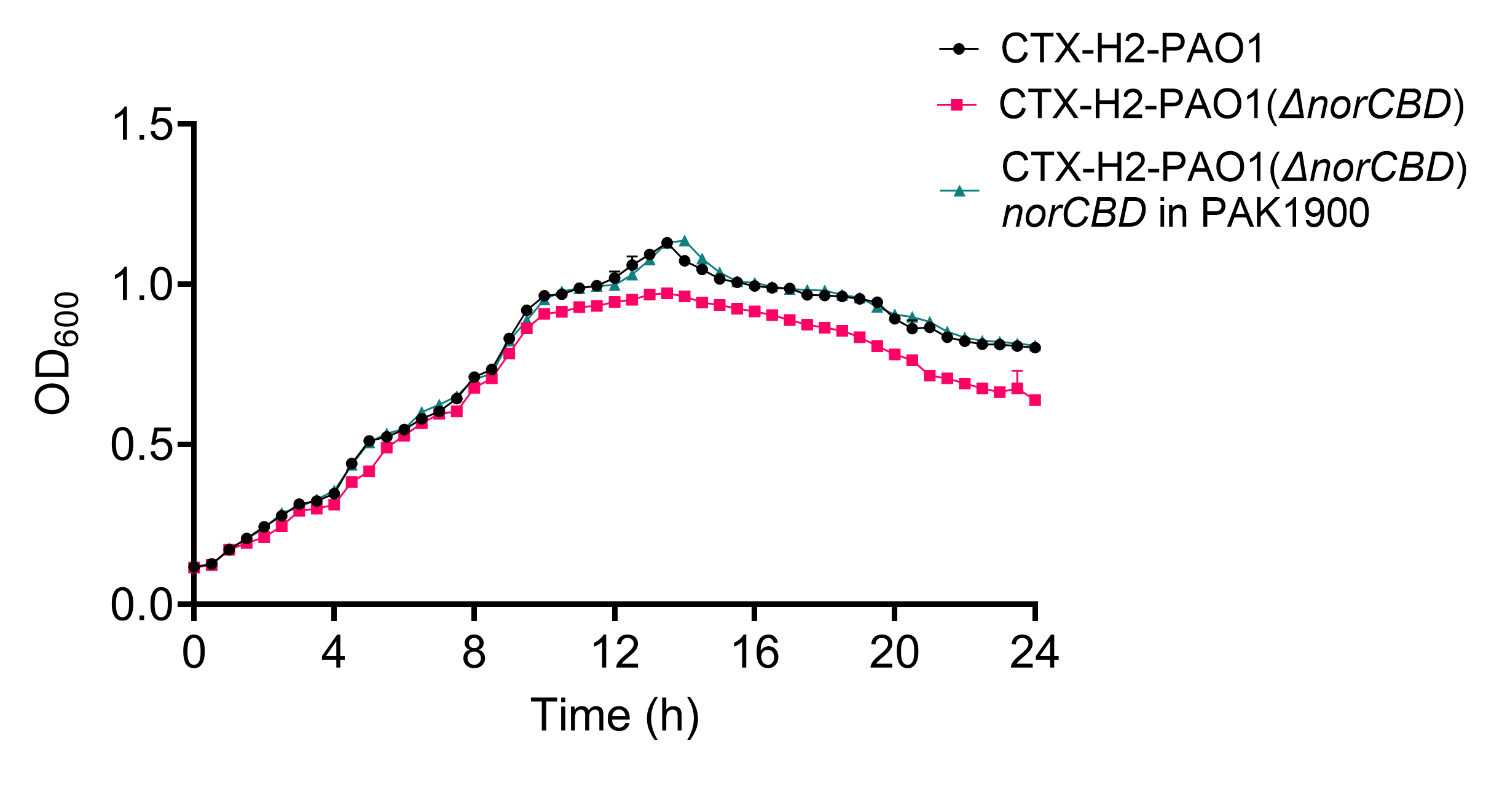


**Supplementary Figure 2.** ***P. aeruginosa* growth (OD_600_) under normoxic conditions.** OD_600_ was measured in wild-type PAO1, PAO1*(ΔnorCBD),* and complementation strains, PAO1*(ΔnorCBD)-*norCBD in PAK1900, for 24 hours under normoxic conditions.


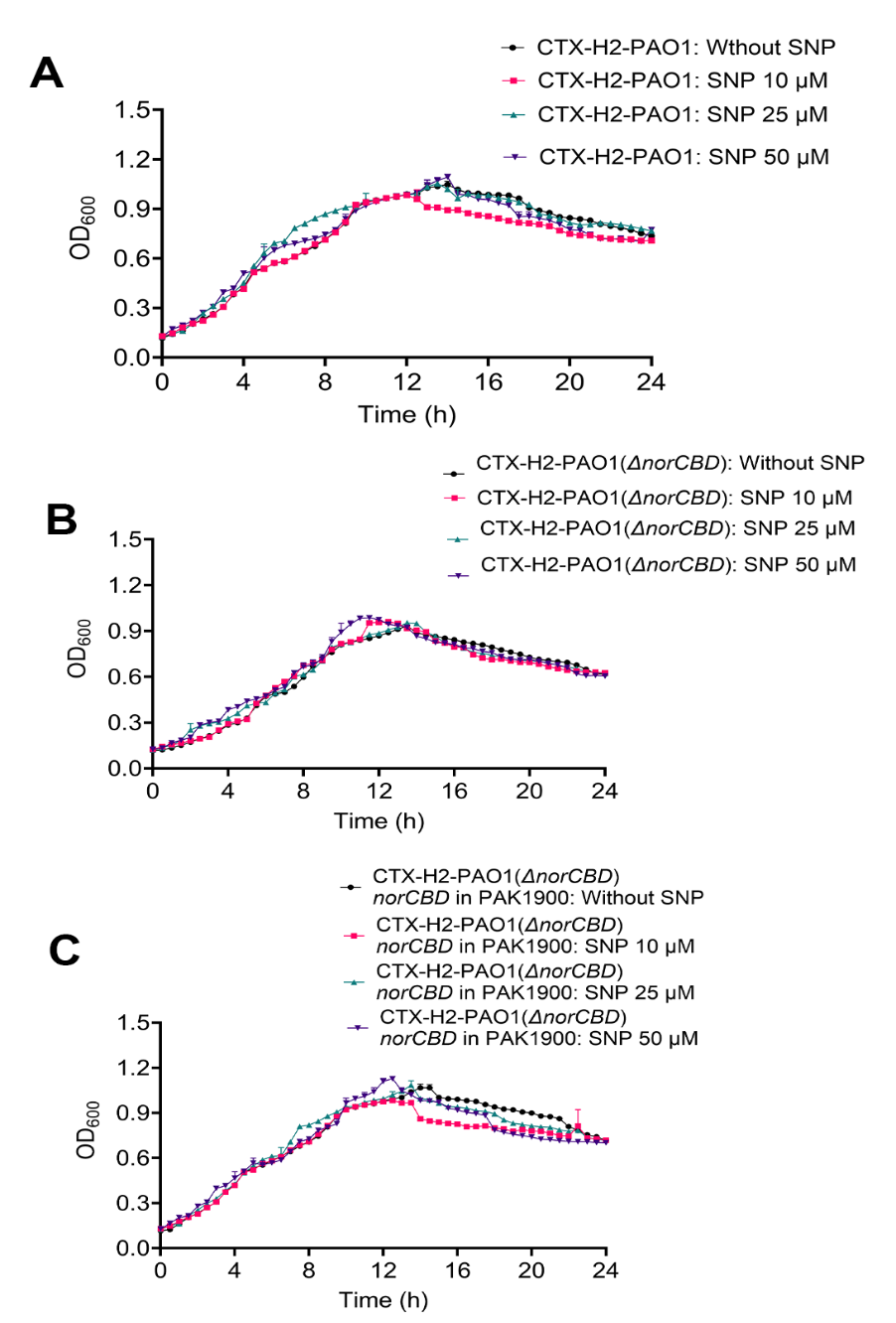


**Supplementary Figure 3 (A, B, C):** ***P. aeruginosa* growth (OD_600_) under normoxic conditions with the addition of SNP.** OD_600_ was measured in wild-type PAO1, PAO1*(ΔnorCBD),* and complementation strains, PAO1*(ΔnorCBD)-*norCBD in PAK1900, for 24 hours, and was monitored with the addition of different concentrations of SNP (10 µM, 25 µM, and 50 µM**)** under normoxic conditions.


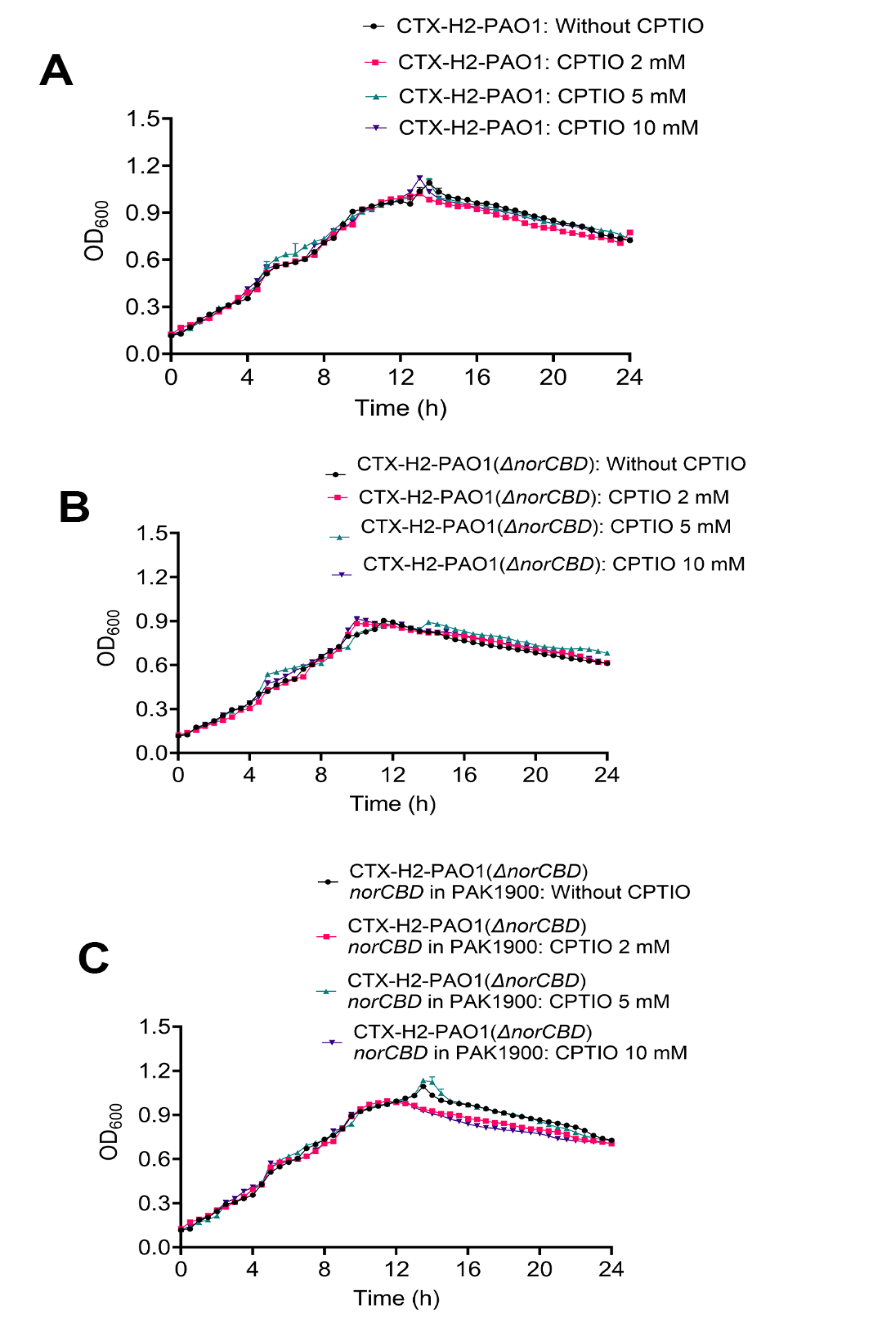


**Supplementary Figure 4 (A, B, C).** ***P. aeruginosa* growth (OD_600_) under normoxic conditions with the addition of CPTIO.** OD_600_ was measured in wild-type PAO1, PAO1*(ΔnorCBD),* and complementation strains, PAO1*(ΔnorCBD)-*norCBD in PAK1900, for 24 hours, and was monitored with the addition of different concentrations of CPTIO (2 mM, 5 mM, and 10 mM) under normoxic conditions.
